# Supplementary material for: Glenn circulation causes early and progressive shunting in a surgical model of pulmonary arteriovenous malformations
Source: Physiol Rep. 2024 Nov 22;12(22):e70123. doi: 10.14814/phy2.70123 (PMC11584281; doi:10.14814/phy2.70123)
Supplement: Supplementary file 1 — Figure S1. [file PHY2-12-e70123-s002.pdf]

**SUPPLEMENTAL MATERIAL**

# Glenn circulation causes early and progressive shunting in a surgical model of pulmonary arteriovenous malformations

Tina Wan, PhD[1,4], Henry Rousseau, BA[1], Carol Mattern, RDCS[1], Madeline Tabor, BS[2], Matthew R. Hodges, PhD[2], Ramani Ramchandran, PhD[3,4], Andrew D. Spearman, MD[1,4]*

1. Department of Pediatrics, Division of Cardiology, Medical College of Wisconsin, Children's Wisconsin, Herma Heart Institute, 9000 West Wisconsin Avenue, Milwaukee, WI 53226
2. Department of Physiology, Medical College of Wisconsin, Children's Wisconsin, 8701 West Watertown Plank Road, Milwaukee, WI 53226
3. Department of Pediatrics, Division of Neonatology, Medical College of Wisconsin, 8701 West Watertown Plank Road, Milwaukee, WI 53226
4. Cardiovascular Center, Medical College of Wisconsin, 8701 West Watertown Plank Road, Milwaukee, WI 53226

**Corresponding author:**

Andrew D. Spearman, MD

Email: aspearman@mcw.edu

Phone: 414.955.2274

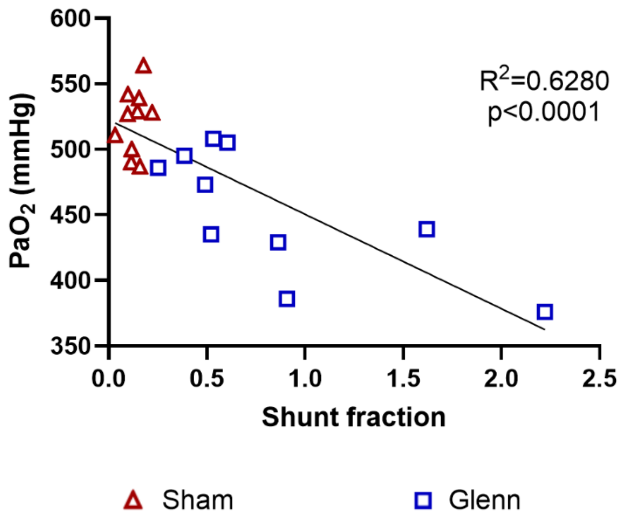


**Supplemental Figure 1: Moderate negative correlation between oxygenation and intrapulmonary shunting of microspheres**. XY scatter plot demonstrates relationship between oxygenation (quantified by arterial blood gas) and shunt fraction with simple linear regression quantifying statistically significant moderate correlation. N=20 biological replicates, including n=10 sham and n=10 Glenn.
