# Supplementary material for: Glenn circulation causes early and progressive shunting in a surgical model of pulmonary arteriovenous malformations
Source: Physiol Rep. 2024 Nov 22;12(22):e70123. doi: 10.14814/phy2.70123 (PMC11584281; doi:10.14814/phy2.70123)
Supplement: Supplementary file 2 — Video S1. [file PHY2-12-e70123-s001.zip › Supplemental Material - Supp Video Legends.pdf]

SUPPLEMENTAL MATERIAL

Glenn circulation causes early and progressive shunting in a surgical model of pulmonary arteriovenous malformations

Tina Wan, PhD[1,4], Henry Rousseau, BA[1], Carol Mattern, RDCS[1], Madeline Tabor, BS[2], Matthew R. Hodges, PhD[2], Ramani Ramchandran, PhD[3,4], Andrew D. Spearman, MD[1,4]*

1. Department of Pediatrics, Division of Cardiology, Medical College of Wisconsin, Children's Wisconsin, Herma Heart Institute, 9000 West Wisconsin Avenue, Milwaukee, WI 53226
2. Department of Physiology, Medical College of Wisconsin, Children's Wisconsin, 8701 West Watertown Plank Road, Milwaukee, WI 53226
3. Department of Pediatrics, Division of Neonatology, Medical College of Wisconsin, 8701 West Watertown Plank Road, Milwaukee, WI 53226
4. Cardiovascular Center, Medical College of Wisconsin, 8701 West Watertown Plank Road, Milwaukee, WI 53226

Corresponding author:

Andrew D. Spearman, MD

Email: aspearman@mcw.edu

Phone: 414.955.2274

Supplemental Video 1: View of the surgical field through a left thoracotomy after retraction of the left lung but before surgical dissection.

Supplemental Video 2: View of the surgical field through a left thoracotomy after completing end-to-end cavopulmonary anastomosis of the left superior vena cava (L-SVC) and left pulmonary artery (LPA).

Supplemental Video 3: Echocardiographic imaging in a modified apical view of a sham rat during a bubble echocardiogram. Agitated saline is visualized opacifying the right ventricle (left side of image) after injection, but agitated saline is not visualized in the left ventricle (right side of image). Lack of agitated saline in the left ventricle indicates a negative bubble study because no bubbles shunted through the lung vasculature into the left ventricle.

Supplemental Video 4: Echocardiographic imaging in a modified apical view of a Glenn rat during a bubble echocardiogram. Agitated saline is visualized opacifying the left ventricle (right side of image) after injection, which indicates a positive bubble study with bubbles shunting through the lung vasculature into the left ventricle. Peak density of bubbles filling the left ventricle indicate a moderately positive bubble echo.

Supplemental Video 5: Echocardiographic imaging in a modified apical view of a Glenn rat during a bubble echocardiogram. Agitated saline is visualized opacifying the left ventricle (right

side of image) after injection, which indicates a positive bubble study with bubbles shunting through the lung vasculature into the left ventricle. Peak density of bubbles filling the left ventricle indicate a trivially positive bubble echo.
